# Routine immunization: an essential but wobbly platform

**DOI:** 10.9745/GHSP-D-13-00122

**Published:** 2013-11-14

**Authors:** Robert Steinglass

**Affiliations:** a John Snow, Inc., Arlington, VA, USA

## Abstract

Despite their vital role, routine immunization programs are taken for granted. Coverage levels are poor in some countries and have stagnated in others, while addition of new vaccines is an additional stressor. We need to strengthen: (1) policy processes, (2) monitoring and evaluation, (3) human resources, (4) regular delivery and supply systems, (5) local political commitment and ownership, (6) involvement of civil society and communities, and (7) sustainable financing. Rebalancing immunization direction and investment is needed.

One-third of the way through the so-called Decade of Vaccines, this is an exciting time for immunization. The World Health Assembly has endorsed a Global Vaccine Action Plan (GVAP),^1^ in which immunization is to be extended as a human right beyond infancy across the entire life cycle. Powerful new life-saving vaccines against some causes of pneumonia and diarrhea, the biggest contributors to child mortality, are being introduced. With a global sense of purpose and vastly increased resources, new partners have entered the vaccine and immunization arena. Anticipating the end of poliomyelitis, some are already scanning the horizon for the next disease to eradicate. However, at this promising moment, the immunization, disease control, and development communities collectively would do well to reflect on past and current directions so as to engage in a genuine debate about the need to restore balance within the realm of immunization.

## ROUTINE IMMUNIZATION NEEDS GREATER ATTENTION

One school of thought holds that investments to achieve broad health system strengthening (HSS) goals will, like the tide, raise all boats and so contribute to the strengthening of routine immunization (RI).^2^ The proponents of this theory use a preferred metric of reduced child mortality from all causes. Another school claims that investments in single-disease control, elimination, or eradication initiatives will strengthen RI and health systems more broadly.^3^ The preferred metric among these thinkers is declining incidence of specific diseases; they argue primarily for investments in disease surveillance, laboratory support, and adverse events monitoring. Proponents on both sides in this long-standing debate, sometimes characterized as horizontal versus vertical approaches, believe strongly in their own path and argue that the funds are never enough, so choices have to be made. Each side claims its own goal is always just around the corner and achievable, if we just stay the course.

Both sides claim to be strengthening RI and make rhetorical claims that RI is a highly valued platform upon which their own ambitious goals partially rest. Rather than advocating increased investment directly into RI strengthening, each school of thought perpetuates a narrative that investments in their own approach obviate the need to invest in RI directly. The sides in this debate sometimes unhelpfully dismiss each other either as “health-content-free reformers” or “disease cowboys.” However, both schools overlook a more balanced, middle path: direct investment in RI strengthening as an integral part of the broader health system to achieve disease reduction goals.

Although responsible for the majority of immunizations given in nearly all countries, RI receives little attention, even from within the immunization community. As children will always be born and therefore always need to be vaccinated, RI programs exist in perpetual need of funds. Lacking strong champions, RI programs cannot generate the resources needed by global partners and ministries of health. Instead, prospective donors are promised that their investments in HSS and especially single disease initiatives will not only accomplish the primary goal, but also will produce any number of positive collateral spin-offs, including the strengthening and improved quality of RI.

Routine immunization receives little attention, even from within the immunization community.

The establishment and maintenance of RI systems suffer from a public image crisis. For some, RI is boring by definition, since it is routine. And if immunization is routine, this suggests a business-as-usual approach, which would be indefensible in the face of unacceptable levels of preventable child mortality. Otherwise, the reasoning goes, if RI was important enough, it would be reframed as a public health emergency, as has polio eradication. In contrast, RI advocates prefer to think that strong and steady investments in RI will win the race, and yet they often struggle to convince donors seeking a more rapid return on investments, in keeping with institutional mandates and short political cycles.

The global immunization community has prioritized improving access to new life-saving vaccines and eradicating or eliminating vaccine-preventable diseases. But the means to achieve these goals rely heavily on a continuously functioning RI program, which often receives only rhetorical attention and scant support.

## ROUTINE IMMUNIZATION IS AN UNDERVALUED WORKHORSE

Powerful new modeling and decision-support tools such as the LiST (Lives Saved Tool)^4^ focus on a potential for future incremental mortality reduction. However, the significant *past* achievements in child mortality reduction, owing to more mature interventions such as immunization, cannot be maintained without continued focus and investment.^5^ With the current deployment of new vaccines against some infections that cause pneumonia and diarrhea, the World Health Organization (WHO) estimates that 29% of deaths among children 1–59 months of age are currently vaccine-preventable.^6^ These new vaccines, as well as future ones being developed for malaria and HIV, will require a strong RI platform to deliver their promise.

Although rarely acknowledged, RI is the workhorse needed to sustain gains from episodic mass immunization campaigns and to prolong intervals between those campaigns. For example, WHO estimates that, of the approximate 12.7 million measles deaths prevented by immunization from 2000 to 2008, 66% (8.4 million) were averted by maintaining RI coverage at the year 2000 level, while an additional 33% (4.3 million) were averted through increases in RI coverage and mass measles immunization campaigns.^7^ Yet despite the importance of RI, the support it requires is not costed in regional and national measles elimination plans, particularly in countries with weaker health systems, leaving these disease-specific plans decidedly unbalanced.

## THE PLIGHT OF ROUTINE IMMUNIZATION

In some of the regions with the poorest performing countries and the greatest need, resources devoted to RI are scarce. For example, over at least the past 5 biennial budgets in the WHO Regional Office for Africa, only approximately 5% of the immunization budget has been directly devoted to RI, and not all of that is mobilized or used. The vast majority (85%) of the WHO/AFRO immunization budget goes to polio eradication.^8^-^11^ Furthermore, the Africa Routine Immunization System Essentials (ARISE) project documented a widespread misperception that the Global Alliance for Vaccines and Immunisation (GAVI) provides major support for immunization service delivery, whereas in fact approximately 90% of their support goes for the purchase of new vaccines.^12^

Because vaccinations can be counted, although not as accurately as desired (as evidenced by disparities between service statistics and many Demographic and Health Survey findings), program performance tends to be regularly measured by coverage levels reported at national and sub-national levels. In recent years, global and regional RI coverage levels have largely stagnated, with large disparities within and among countries. In the lower-performing regions such as Africa and Southeast Asia and in a large number of countries within every region, infant coverage with the third dose of DTP (diphtheria-tetanus-pertussis) and the first dose of measles vaccines, both given through the routine system, has either declined or stagnated at between 70% and 80% in each of the past 5 years.^13^-^14^ And the global number of children not fully immunized with the third dose of DTP-containing vaccines—more than 20 million now—has remained largely unchanged over the past 5 years.^6^

Global and regional routine immunization coverage levels have largely stagnated, with large disparities within and among countries.

## ROUTINE IMMUNIZATION IS A DEVELOPMENT CHALLENGE REQUIRING A SYSTEMS APPROACH

The challenges facing RI are great, and immunization coverage must not be the only consideration. Before exposure to disease, infants and other individuals must be routinely reached with potent vaccines in a safe, effective, and affordable way and with adequate service quality, so that they will want to return to complete all their doses. Achieving this within the overarching health system must be recognized as a development challenge, broader than a disease-control challenge. A steady marathon, not a sprint to some short-term finish line, is required.

Strengthening routine immunization within the overarching health system must be recognized as a development challenge.

Retaining the trust of families and communities is an old challenge that requires systematic effort to ensure the predictability and quality of services. Families need information and counseling to become aware of and accept immunization services. Families must know where and when to come for immunization and to bring vaccination cards. They must be treated affably and respectfully by health workers.

Building a strong RI program as an integral part of the health system is a development challenge that requires a systems approach. As illustrated in the figure, a cohesive, well-functioning immunization program includes many components, all of which dynamically interact to influence the accessibility, availability, acceptability, and affordability of services, with a desired result of continuous coverage, improved service quality, equity, and sustained disease control. Immunization programs require more than just a supply of vaccines and surveillance, which are often the dominant focus of donors. But many countries in Africa and Asia do not receive a balanced and multidisciplinary package of technical support from partners across the many interconnected components. Instead, certain key RI program components (such as supply chain management, program communication, community partnerships, supportive supervision, local use of performance data, and financial management) are consistently neglected for a variety of reasons, including the institutional preferences of partner agencies, formulaic top-down prescriptions, and the absence of a learning culture to identify and spread innovative approaches.

Many countries in Africa and Asia do not receive a balanced and multidisciplinary package of technical support from partners across the many interconnected components.

**Figure.**
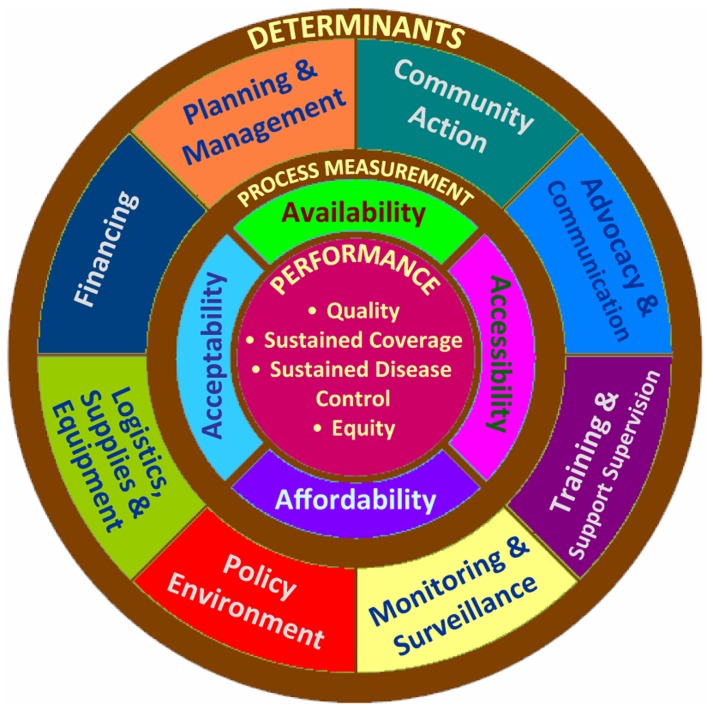
Conceptual Framework: Immunization Sub-System Source: Adapted from reference 15.

Strengthening RI system performance and monitoring, as part of a functioning health system, is the foundation to achieving immunization goals. Yet, despite their relative maturity, RI programs in many countries have become increasingly fragile, even as they face new challenges, as evidenced by a plethora of national immunization reviews, vaccine management assessments, and reviews following the introduction of new vaccines. Despite known gaps and the introduction of new vaccines, the vast majority of low-performing countries receive no technical support to strengthen capacity for immunization supply chain management. Most WHO regions have no officer looking after cold chain and logistics. As with the cold chain, strengthened capacity through constant attention, maintenance, and repair is needed for sustained, effective, and efficient performance of the entire program. The substantial accomplishment of RI programs in the past does not guarantee success in the future.

## HOW CAN ROUTINE IMMUNIZATION BE STRENGTHENED?

As identified by one of the Decade of Vaccines working groups,^16^ co-led by the author, key approaches to strengthen performance of RI systems and monitoring must include:

- **Strengthening the structure and processes for developing immunization policy, strategies, and best practices.** Policies and strategies for public health should be developed nationally, taking into account national health priorities and capacities, rather than by top-down implementation of global and regional recommendations. This is essential to promote greater country ownership and commitment to the program and to reduce external dependence.
- **Improving systems and tools for generating evidence, monitoring program performance, and the use of data for action.** Strengthening the capacity for monitoring and surveillance is required to improve use and quality of data to inform policies and strategies, for planning at all levels, and for documenting the impact of the program. The use of coverage and management data also needs strengthening at sub-national levels to locally identify and correct problems.
- **Training, deploying, supporting, and supervising adequate human resources for program management and implementation.** Sufficient numbers of competent health workers, equitably deployed and supported by strong health systems, are required to manage and implement immunization programs. New insights must be applied into what works in planning, managing, educating, and supporting health workers, both formal and informal. New approaches are needed to support learning through pre- and in-service education and to address lack of motivation and underperformance.
- **Building, maintaining, and sustaining regular immunization delivery and supply systems.** Cold chain and logistics systems, which have been established, strengthened, and maintained over the past 30 years as the backbone of immunization programs, must be revitalized to face new challenges (such as new, more costly, more bulky vaccines) and to take advantage of technological advances. Today, more than ever before, managers must be able to maintain lower stock levels, reduce wastage, accurately forecast vaccine requirements, and prevent equipment breakdowns or malfunctions so that target populations can access and make use of high-quality and safe immunization services.
- **Promoting greater ownership, political commitment, accountability, and self-reliance of immunization programs at all levels.** Political commitment, good stewardship, and broad political engagement at national and sub-national levels are required to own and support the immunization program. Given the numbers of other important stakeholders, ranging from religious and community leaders to civil society organizations, to parents and caretakers, efforts to stimulate and sustain societal commitment are also needed. This commitment should contribute to increased accountability and country ownership of immunization programs, thereby leading to greater cooperation, participation, and ultimately even increased government funding.
- **Broadening the engagement of civil society and communities.** Civil society has an essential—but underappreciated—role to play in the full range of immunization activities, from policy development through resource mobilization and accountability, service delivery, reaching underserved communities, demand creation, surveillance, operational research, and monitoring and evaluation. Better engagement of civil society will be necessary to ensure strong immunization and other health programs.
- **Achieving sustainable immunization financing and sound financial management.** Poor financial management and heavy dependence on donors are interrelated problems. Consequently, the efficiency of programs is unknown, and there is little or no budget oversight or accountability. Budgeting reforms, improved inter-ministerial financial management, and long-term innovative financing mechanisms must be addressed together so that governments and other domestic stakeholders have the incentive to invest more in immunization and reach sustainable financing goals. More advocacy and oversight from parliaments, sub-national officials, community service organizations, and other influential groups will add strong new incentives.

Many of these approaches have begun to receive new attention.

## PROMISING DEVELOPMENTS

In addition, there have been some promising recent developments among global partners. The Bill & Melinda Gates Foundation developed a RI strategy in 2012 to identify and focus on some of the unaddressed challenges, particularly the need to improve data quality and use, as well as immunization supply chain logistics necessary to protect investment in expensive new vaccines.

GAVI has recognized that its investment model, in which 90% of its funds are spent on vaccine procurement, must be complemented with more focused support for HSS in which RI takes center stage. There is also greater recognition that improving “access” to vaccines—primarily focusing on their development, supply, and financing—must be accompanied by a greater focus on strengthening the delivery, quality, timeliness, and use of immunization services.

WHO and the United Nations Children's Fund (UNICEF) manage a Vaccine Presentation and Packaging Advisory Group, in which industry and public health officials come together to identify desirable characteristics of future vaccines. WHO has revamped its vaccine pre-qualification process to highlight characteristics of vaccines that are programmatically most suitable for use in less-developed countries.

UNICEF is rightly stressing the need in all of its programming to reach underserved populations—for example, the urban poor, remote communities, marginalized groups—and to address equity gaps. Current global support for “universal health coverage” provides a mandate for “universal immunization coverage,” and UNICEF's advocacy will be needed to ensure that immunization is vigorously included in such efforts.

## IMPLEMENT ROUTINE IMMUNIZATION MORE SMARTLY

Despite these encouraging changes in partners' focus, RI programs must still learn to adapt in order to overcome persistent challenges and seize new opportunities. RI programs should engage better with broader HSS efforts, such as using information technology more effectively, to ensure that immunization is well-addressed.

What has worked in the past may have reached its limits. In their impatience to rapidly improve coverage by overcoming immediate obstacles, as opposed to strengthening the overall RI system, partners sometimes promote unsustainable workarounds—often in the name of integrated service delivery, during which vitamin A supplements, bed nets, and other services are offered along with immunization. Periodic intensification of RI, for example, through semi-annual Child Health Days or Weeks, can be effective if they actually reach additional underserved populations with immunization services, but they must be deliberately planned and executed to avoid undermining the ability of the RI system to function for the remaining 50 weeks of the year.^17^ For example, at the end of intensified days or week-long campaigns, especially if new service delivery points have been temporarily created for the event, health workers then face difficulty integrating health records and screening the immunization status of individual children, who must continue to receive the remaining doses in the series.

More focus is needed in specific areas. For example, operationalizing the WHO/UNICEF Reaching Every District (RED)^18^ approach to reach the underserved deserves greater attention and better quality efforts from technical partners. Because RI services can be scheduled, they can be better planned, managed, supervised, monitored, and linked with communities. The RED approach is a common-sense package for doing that, but in too many countries “doing RED” has become a meaningless mantra, reduced to a one-off training exercise focusing only on the micro-planning component of the approach.

The immunization community also must move beyond the never-ending cycle of gap/barrier/bottleneck analyses that launch every initiative, identifying long lists of problems (for example, “cold chain broken,” “supervision weak”) and making unhelpful, anodyne recommendations (“fix the cold chain,” “strengthen supervision”). But what works? What drives good performance? And under what conditions do innovations and good practices emerge, take root, and spread? What pre-conditions should be cultivated to encourage the development of promising approaches and innovations? Immunization programs at all levels must do a better job of capturing and accelerating learning about what works, how it works, and in what contexts it works—so as to spread promising practices more rapidly.^19^ Using implementation research approaches such as assets-based, appreciative inquiry and a mix of quantitative and qualitative methods, immunization programs need to identify positive deviants (those that are performing well in spite of common constraints) among district managers, health facility staff, and community leaders who can serve as examples to others facing similar challenges.^20^

Immunization programs must do a better job of capturing and accelerating learning about what works, how it works, and in what contexts it works.

## WHAT CAN BE LEARNED FROM THE POLIO ERADICATION LEGACY?

Polio certainly must be eradicated, and soon, but this message needs to be better nuanced, without overstating what collateral benefits the polio model and assets contribute to RI strengthening. A one-time sprint to the polio eradication finish line differs from the marathon approach required to affordably and sustainably develop and strengthen RI programs. The operational strategies employed by the Global Polio Eradication Initiative (GPEI)—such as well-funded armies of “volunteers” going door to door to vaccinate children—are very resource-intensive in terms of money and staff and do not need to be sustained. For GPEI, the end justifies the means—but those means can distort RI programs and health services more generally. With ample funding and staff, the GPEI understandably does end-runs around the health system. These shortcuts work for the GPEI but not for RI programs, which must determine how to overcome systemic barriers and work affordably through the health services. Contrary to the GPEI, the RI program must reach individual children in a timely way with all appropriate age- and dose-specific vaccines through reliable and good-quality services, year after year. The planned introduction of the injectable inactivated polio vaccine over the coming few years in more than 100 countries, as part of the so-called end-game strategy,^3^ has some potential to increase the focus on RI strengthening.

## A BALANCED WAY FORWARD

A robust RI program, functioning within the health system, is required to achieve ambitious goals and sustain them. These goals include disease elimination and eradication, smooth introduction of new vaccines across an expanded life cycle, increased immunization coverage, greater equity, and reduced mortality to achieve the Millennium Development Goals. To be successful, the global community must recognize that the fundamental platform—the routine immunization system itself—must be directly supported and reinforced.

The immunization program must be re-balanced to consolidate and sustain its gains and to complete the task of polio eradication, rather than prematurely embarking on new, overambitious goals. The global health and development community should insist on a moratorium on adopting any more disease-eradication goals without a broader and vigorous public debate, evidence identifying programmatic and financial opportunity costs in advance, a determination that the benefits outweigh the risks and costs in all populations, and a system in place to hold these initiatives more accountable for specifically strengthening the RI system and the health system more broadly. In the near-term, the immunization community must endeavor to advocate the eradication of polio without overstating its so-called “legacy” for RI.

New vaccine introduction, polio eradication, measles elimination, and tetanus elimination efforts must be used more strategically and deliberately to overcome persistent system challenges, optimize positive spinoffs, and mitigate negative ones. Judging from experience, this does not happen automatically. What will it take to do so?

The immunization community must aim not only to increase but also to rebalance current and future investments, so as to sustainably overcome the recognized but still chronic weaknesses in RI programs. This will require mobilizing and sustaining not only political will but also social will at all levels of government to meet the recurring operational costs of programs over time—not just focusing on the next externally driven event (for example, mass campaign or new vaccine launch) on the horizon.

The immunization community must aim not only to increase but also to rebalance current and future investments.

The immunization community must assure that essential preventive services such as RI continue to work in the context of health sector reform, whether it involves integration, decentralization, or privatization. Constantly scanning the horizon to identify opportunities and threats, making necessary adaptations, and seeking continued investment are necessary. In addition, health services should do a better job of using the RI system as a vehicle to scale up other population-based interventions.

To overcome stagnation and improve performance, the immunization community must create opportunities for countries, districts, and health facilities to learn from each other and to better identify and accelerate the spread of good practices. The ultimate innovation could be to create a stimulating learning culture that seeks to do this. A culture of learning is more likely to emerge when it includes multiple perspectives, diverse disciplines, and broad partnerships, and when staff members have an opportunity to learn from peers working on the same problems.^19^

A culture of learning is more likely to emerge when it includes multiple perspectives, diverse disciplines, and broad partnerships.

Vaccines offer unprecedented promise to reduce incalculable human misery, but only by creating and maintaining a vigorous routine immunization platform will this come to pass.
